# Gibberellin Biosynthetic Deficiency Is Responsible for Maize Dominant Dwarf11 (*D11*) Mutant Phenotype: Physiological and Transcriptomic Evidence

**DOI:** 10.1371/journal.pone.0066466

**Published:** 2013-06-12

**Authors:** Yijun Wang, Dexiang Deng, Haidong Ding, Xiangming Xu, Rong Zhang, Suxin Wang, Yunlong Bian, Zhitong Yin, Yao Chen

**Affiliations:** 1 Key Laboratory of Crop Genetics and Physiology of Jiangsu Province, Key Laboratory of Plant Functional Genomics of Ministry of Education, College of Agriculture, Yangzhou University, Yangzhou, China; 2 Key Laboratory of Crop Genetics and Physiology of Jiangsu Province, College of Bioscience and Biotechnology, Yangzhou University, Yangzhou, China; China Agricultural University, China

## Abstract

Dwarf stature is introduced to improve lodging resistance and harvest index in crop production. In many crops including maize, mining and application of novel dwarf genes are urgent to overcome genetic bottleneck and vulnerability during breeding improvement. Here we report the characterization and expression profiling analysis of a newly identified maize dwarf mutant *Dwarf11* (*D11*). The *D11* displays severely developmental abnormalities and is controlled by a dominant Mendelian factor. The *D11* seedlings responds to both GA_3_ and paclobutrazol (PAC) application, suggesting that dwarf phenotype of *D11* is caused by GA biosynthesis instead of GA signaling deficiency. In contrast, two well-characterized maize dominant dwarf plants *D8* and *D9* are all insensitive to exogenous GA_3_ stimulation. Additionally, sequence variation of *D8* and *D9* genes was not identified in the *D11* mutant. Microarray and qRT-PCR analysis results demonstrated that transcripts encoding GA biosynthetic and catabolic enzymes *ent*-kaurenoic acid oxidase (KAO), GA 20-oxidase (GA20ox), and GA 2-oxidase (GA2ox) are up-regulated in *D11*. Our results lay a foundation for the following *D11* gene cloning and functional characterization. Moreover, results presented here may aid in crops molecular improvement and breeding, especially breeding of crops with plant height ideotypes.

## Introduction

The well-known ‘Green revolution’ highlighted in the late 1960s and increased food crops yield worldwide by incorporating semi-dwarf traits combined with other agricultural practices, such as fertilizer and pesticide utilization, irrigation equipment installation. The technical advance of ‘Green revolution’ mainly attributes to the development of ‘high-yielding varieties’ (HYVs) which show high capacity of nitrogen assimilation and tend to lodge. Dwarf traits were introduced to improve lodging resistance in this case. During the ‘Green revolution’, novel dwarf rice and wheat cultivars with increased yield were developed. Semi-dwarf variety IR8 (also known as ‘Miracle Rice’), the first widely used HYV of International Rice Research Institute (IRRI), was bred by a Chinese dwarf variety ‘Dee-geo-woo-gen’ crossed with an Indonesian high-yielding variety ‘Peta’. The Japanese dwarf wheat cultivar ‘Norin 10’ was adopted to breed wheat HYVs [1]. With the development of diverse omic-based technology, the genes of ‘Green revolution’ *semidwarf1* (*sd1*) and *Reduced height* (*Rht*) were isolated and characterized. Rice *sd1* encodes a defective gibberellin (GA) biosynthetic enzyme GA 20-oxidase (GA20ox). Wheat *Rht* has been proved to be involved in GA signaling cascade. Both *sd1* and *Rht* genes participate in GA homeostasis [2].

Maize dwarf trait research could be traced back to Emmerson's work [3]. Over the past decades, a large amount of maize dwarf mutants have been identified and characterized. Detailed information of maize dwarf plants has been deposited in the MaizeGDB phenotypic database (http://www.maizegdb.org/phenotype.php). Maize dwarf mutants are primarily classified as GA-sensitive and GA-insensitive types based on their responses to exogenous GA application. Four recessive dwarf mutants *dwarf*-1 (*d1*), *d2*, *d3*, and *d5*, together with one dominant dwarf mutant *D8-1023* belong to the GA-responsive group [4], [5]. Two well-characterized maize dominant dwarf plants *Dwarf8* (*D8*) and *D9* are insensitive to exogenous GA stimulation [6]. The change of DELLA domains in maize D8 and D9 proteins causes dwarf phenotype [7], [8]. *D8-1023*, allele of *D8*, produces dwarf plants by altering the VHYNP domain instead of DELLA domain [5]. A combined linkage and association analysis technique revealed that *d8* functions not only in plant height control but flowering time determinant [9]. However, recent reanalysis of *d8* locus by newly developed model indicated that *d8* has minor effect on flowering time [10]. Besides, candidate genes regulating maize height were unraveled by genome wide association studies (GWAS) technique [11]. Recently, *GA3ox2* was positionally cloned and proved to be responsible for maize height determinant [12].

Many plant height-regulating genes have been isolated in other cereal crops. Barley *Sln1*, wheat *Rht*, rice *slender rice 1* (*slr1*), and maize *d8* are orthologs of *Arabidopsis Gibberellin Insensitive* (*GAI*) and *REPRESSOR OF ga1-3* (*RGA*) genes, which encode DELLA proteins and participate in GA signaling [7], [13], [14]. One member of APETALA2 (AP2)/Ethylene-Responsive Element Binding Factor (ERF) family significantly affects rice internode elongation by down-regulating of GA concentrations [15]. In addition to plant hormone GA, other phytohormones, such as auxin (Aux) and brassinosteroid (BR), modulate plant height. Maize dwarf plant with compact stems *brachytic2* (*br2*) and sorghum counterpart *dwarf3* (*dw3*) show polar auxin transport abnormalities in the stalk [16]. The BR metabolism pathway and signaling perception also relate to rice, maize, and wheat height control [17]–[20]. Besides, a recent research showed that an AP2-like gene mutation results in maize internode length decrease [21]. Plant cytochrome P450 member could also modulate rice height by influencing cell elongation [22].

Plant height, an important agronomic trait, is vital in shaping plant architecture and then ultimately affecting crop yield. Dissection of molecular bases underlying plant height is beneficial to both basic and applied research. During the past, a large number of plant height QTL/genes have been identified (http://www.gramene.org/qtl/), which is helpful for elucidating the genetic mechanisms of plant height determinant. Of note, dwarf mutants are informative for the plant height research. Herein, we report physiological and transcriptomic analysis of a maize dominant dwarf plant, which was temporarily termed *Dwarf11* (*D11*) in our work. Results presented here provide insights into the biological network of plant height control and pave the way for the following *D11* gene cloning and functional characterization.

## Results

### Characterization of maize *D11* mutant

Maize inbred line Mo17 was pollinated with maize inbred line HN06 to generate F_1_ progeny. In F_1_ population, the *D11* mutant was identified. Due to abnormal inflorescence, it failed to self-pollinate *D11*. To preserve mutant phenotype, we continuously backcrossed *D11* (as donor parent) with three maize inbred lines Mo17, B73, and W22. Finally, three advanced backcross populations BC_6_F_1_ with different nuclear background were developed.

**Figure 1:**
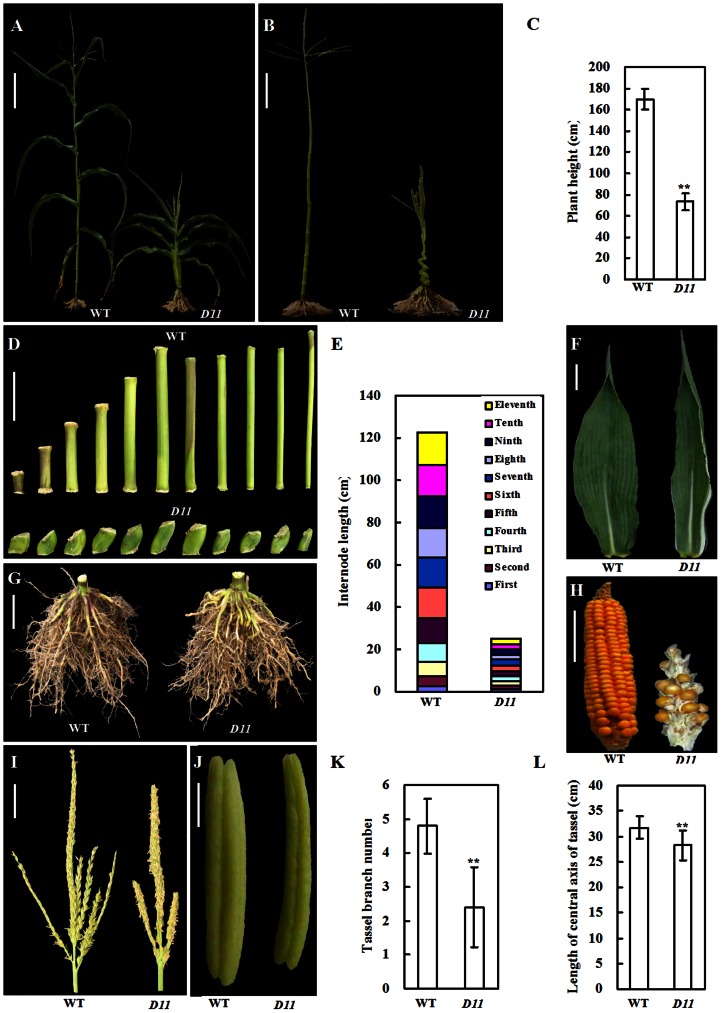
Gross morphology of maize *D11* mutant. (**A**) Phenotype of *D11* and wild type (WT). Bar  = 20 cm. (**B**) Whole plant. To get snapshot of internodes arrangement, leaves and spike were removed manually. Bar  = 20 cm. (**C**) Plant height. (**D**) Internodes. Bar  = 5 cm. (**E**) Internode length. (**F**) Leaf. Leaves of *D11* are slender, dark green, slightly-rolled, and with white margins. Bar  = 5 cm. (**G**) Roots. Aerial roots of *D11* display more sturdy. Bar  = 5 cm. (**H**) Spike. Spike of *D11* degenerates severely. Bar  = 5 cm. (**I**) Tassel. Bar  = 5 cm. (**J**) Anther. Anthers of *D11* are short and thin. Bar  = 1 mm. (**K**) Tassel branch number. (**L**) Length of central axis of tassel. In figures (C), (E), (K), and (L), data are mean ±SD (*n* = 30). Double asterisks denote significant difference at P≤0.01 level compared with the wild type by Student's *t* test.

The *D11* mutant displayed severely developmental abnormalities, such as shortened internodes, white leaf margins, multiple degenerated spikes. The *D11* plant with Mo17 nuclear background was showed in Figure 1. The *D11* mutant was about 73.5 cm tall, which was less than half of wild-type plant (169.8 cm) (Figures 1A–1C). Dwarf phenotype of *D11* mainly attributed to shortened internodes, instead of internodes number decrease. Moreover, eleven internodes of *D11* shortened evenly (Figures 1D and 1E). The *D11* mutant had more leaves. Compared with wild-type plant, leaves of *D11* were slender, dark green, and slightly-rolled. Additionally, leaves of *D11* possessed white margins (Figures 1F and S1). Compared with wild-type, aerial roots of *D11* were more sturdy (Figure 1G). As *D11* displayed multiple degenerated spikes adjacent to the tassel, it was difficult to self-pollinate the mutant plant. In open-pollination conditions, some spikes were partly fertile, others were absolutely barren (Figure 1H). The fertility of *D11* male inflorescence was normal, although anther size, branch number, and length of central spikes of *D11* tassels were largely reduced (Figures 1I–1L). Besides, pollens shed from anthers in the central spikes of *D11* were about two days earlier than shed in wild-type plant.

### Genetic behavior of maize *D11* gene

As the *D11* mutant has abnormal female inflorescence, it was difficult to generate progeny derived from *D11* by self-pollination. Thus, three advanced backcross populations were developed using *D11* as donor parent. To investigate the genetic behavior of *D11* gene, segregation of plant height in three backcross populations with different nuclear background (Mo17, B73, and W22) were analyzed in various years and regions. Results showed that the *D11* mutant phenotype was stable in different years and growth conditions. Moreover, the *D11* mutant phenotype appeared stably regardless of nuclear background. In backcross populations, the ratio of dwarf and normal height plants was 1∶1 which fit closely the expected segregation ratio. Progeny derived from self-pollination of normal height plants was all with normal height (Table S1). Evidence from segregation ratios in backcross and self-pollination populations, together with the phenomenon that *D11* mutant phenotype was firstly observed in F_1_ generation, demonstrated that *D11* inherits as a dominant Mendelian factor.

### Sequence variation in maize *D11* mutant

Two maize dominant dwarf genes *D8* and *D9* have been cloned. To test whether *D11* is allelic to *D8* or *D9*, we dissected sequence polymorphisms between wild-type and *D11* plants. Two pairs of primers were designed on 5′ and 3′ untranslated regions (UTRs) of *d8* and *d9* (Table S2) and adopted to amplify wild-type and *D11* templates (Figure S2). Sequencing of target PCR products showed that there were no sequence polymorphisms between wild-type and *D11* plants, implying that *D11* is not allelic to *D8* and *D9*.

### Response of maize *D11* mutant to exogenous GA_3_ and PAC stimulation

**Figure 2:**
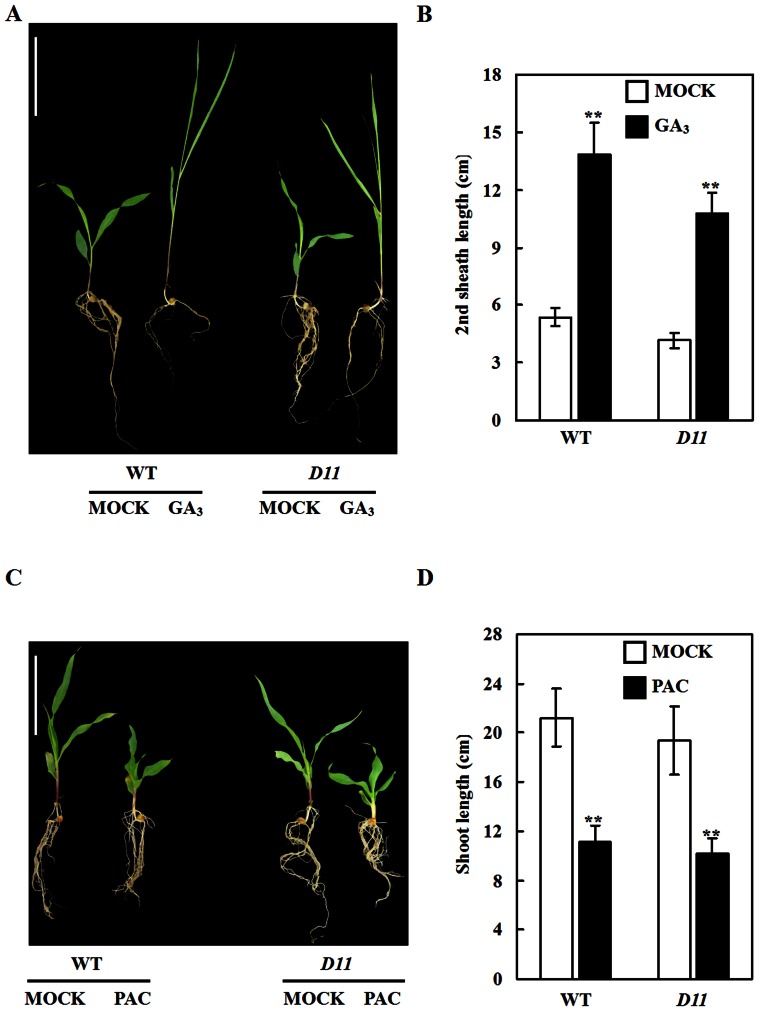
Response of maize *D11* mutant to GA_3_ and PAC application. (**A**) Seedlings of WT and *D11* when treated with a 10^−4^ M GA_3_ solution. Bar  = 10 cm. (**B**) The second leaf sheath length of WT and *D11* (*n* = 35) when treated with a 10^−4^ M GA_3_ solution. (**C**) Seedlings of WT and *D11* when treated with a 10^−4^ M PAC solution. Bar  = 10 cm. (**D**) Shoot length of WT and *D11* (*n* = 40) when treated with a 10^−4^ M PAC solution. In figures (B) and (D), data are mean ±SD. Double asterisks indicate significant difference at P≤0.01 level compared with untreated samples by Student's *t* test.

Two maize dominant dwarf plants *D8* and *D9* are all insensitive to exogenous GA_3_ application. We investigated responses of *D11* to GA_3_ stimulation. When treated with a 10^−4^ M GA_3_ solution, shoot, coleoptile, the first leaf blade, the first leaf sheath, and the second leaf blade length were significantly increased in WT and *D11* (Figures 2A, 2B, and S3). Moreover, GA biosynthesis inhibitor PAC was applied to WT and *D11* seedlings. Results showed that shoot elongation in WT and *D11* was significantly inhibited when treated with a 10^−4^ M PAC solution (Figures 2C and 2D). The *D11* mutant responded to both GA_3_ and PAC stimulation, suggesting that dwarf phenotype of *D11* is caused by GA biosynthesis instead of GA signaling deficiency.

To our knowledge, there are four maize dominant dwarf mutants (*D8*, *D8-1023*, *D9*, and *Dt*) which have been publicly reported. Features of these four maize dominant dwarf plants including *D11* were described in Table 1.

**Table 1:** Snapshot of five maize dominant dwarf plants.

Name	Leaf	Tillering	Floral organ	GA sensitivity	Chromosome	Gene cloning or not	Source
*D8*	Narrow, dark green	√	Andromonoecious	Insensitivity	1	√	[9], [23]
*D8-1023*	Broad, dark green, pursy	√	Andromonoecious	Sensitivity	1	√	[5]
*D9*	Narrow, dark green	√	Normal	Insensitivity	5	√	[8], [23]
*Dt*	N.A.	N.A.	N.A.	Sensitivity	10	×	[24], [25]
*D11*	Narrow, dark green, rolled	×	Abnormal	Sensitivity	2	×	[26], this study

N.A. indicates not available.

### Differentially expressed genes (DEGs) in response to *D11* mutant phenotype

**Figure 3:**
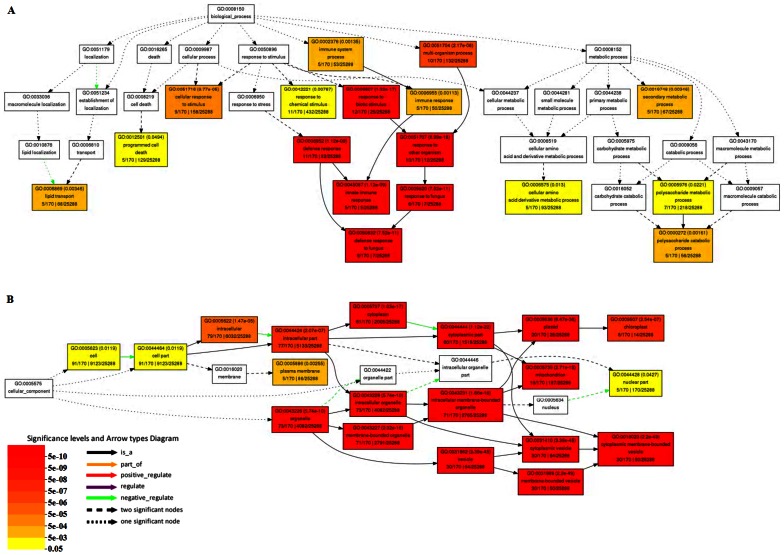
GO clustering of up-regulated DEGs. (**A**) GO enrichment analysis according to GO catalogue (GO:0008150 biological process). (**B**) GO enrichment analysis according to GO catalogue (GO:0005575 cellular component).

There were a total of 17,734 probes which could be detected both in WT and *D11*. In this study, only transcripts with fold change greater than five were considered as DEGs. Finally, 285 DEGs were identified, including 220 up-regulated genes and 65 down-regulated genes (Table S3). To investigate functional characterization of DEGs, up-regulated and down-regulated DEGs were separately subjected to GO enrichment analysis. Detailed information of GO clustering results was presented in Figure 3 and Figure S4.

**Figure 4:**
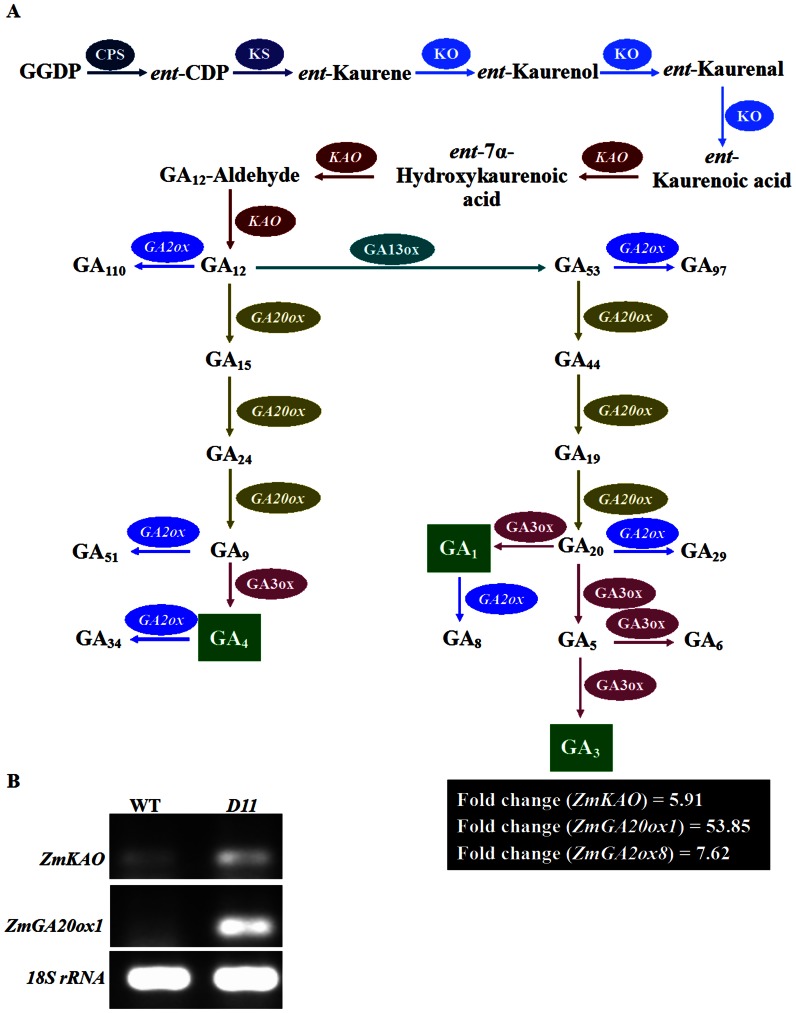
DEGs involved in GA biosynthesis and catabolism. (**A**) GA biosynthesis and catabolism pathways were briefly diagramed. Transcripts encoding maize GA biosynthetic and catabolic enzymes ZmKAO, ZmGA20ox1, and ZmGA2ox8 are up-regulated in *D11*. (**B**) Semi-qRT-PCR validation of elevated transcripts *ZmKAO* and *ZmGA20ox1*. The *18S rRNA* gene was used as an internal control.

The *D11* mutant is characteristic of dwarfism and responds to both GA_3_ and PAC stimulation. To this end, we specifically surveyed DEGs related to phytohormone GA biosynthesis and catabolism. Transcript encoding KAO which is responsible for precursor GA_12_ biosynthesis was up-regulated in *D11*. Expression of *GA20ox1* which is required for the biosynthesis of active GAs from precursor GA_12_ was highly elevated in *D11*. Similarly, transcription of *GA2ox8* which is involved in GA deactivation was relatively high in *D11* (Figures 4A and 4B).

## Discussion

### Possible relationship between plant hormone GA and *D11* mutant phenotype

Plant hormones GA, BR, Aux together with other modulators form an elaborate network to finely trigger plant stature in nature [27]. Especially for phytohormone GA, there is ample evidence supporting its role in plant height control. Two well-characterized maize dominant dwarf plants *D8* and *D9* all belong to the GA-insensitive group, whose dwarf phenotype is caused by GA signaling instead of GA biosynthesis deficiency [6]. Contrarily, physiological analysis in our work demonstrated that maize dominant dwarf plant *D11* responds to exogenous GA_3_ application and is a GA-sensitive mutant.

The GA metabolism pathway has been unraveled [28]. In this study, we investigated the expression patterns of GA metabolic enzyme genes in *D11*. Transcripts encoding GA metabolic enzymes KAO, GA20ox1, and GA2ox8 are all up-regulated in *D11*. Elevated transcripts of *KAO* and *GA20ox1* were further validated by qRT-PCR method (Figure 4). Up-regulation of transcripts encoding GA metabolic enzymes in GA-deficient mutants has been reported previously. In *Arabidopsis* GA biosynthesis-deficient mutant *dwarf and delayed-flowering 1* (*ddf1*), transcripts of three *GA20ox* genes *AtGA20ox1*, *−2*, and *−3* are elevated [29]. Similarly, the expression levels of *OsGA20ox2*, *−4*, and *OsGA2ox6* are increased in rice dominant dwarf and GA-deficient mutant H032 [30]. Up-regulation of transcripts encoding GA metabolic enzymes in GA-deficient mutants may be necessary for GA homeostasis throughout the life cycle of plants. Positive feedback mechanisms may account for this regulatory loop [29].

Plant hormone GA plays a vital role in flower organ development [31]. Systematic analysis of *Arabidopsis GA20ox* genes demonstrated that *AtGA20ox1*, −*2*, and −*3* have significant effects on floral organ growth and anther development [32]. The *D11* mutant displays severe inflorescence abnormalities, characteristic of slender tassel and degenerate spikes (Figures 1H–1L). Overdose of *GA20ox1* gene revealed by microarray and qRT-PCR in our work (Figure 4) may be partly responsible for abnormal inflorescence development in *D11*. Undoubtedly, further physiological, genetic, and developmental data should be collected to test this hypothesis.

Phytohormone GA promotes chloroplast biogenesis through controlling leaf mesophyll cell expansion. In *Arabidopsis* and rice GA-deficient mutants, chloroplast division is largely inhibited. Transcript levels of genes related to chloroplast division are also significantly decreased in GA-deficient mutants [33]. Physiological and transcriptomic analysis in this study indicated that *D11* has defects in GA metabolism. Additionally, *D11* is with white leaf margins (Figures 1F and S1). The relationship between GA deficiency and albino phenotype of *D11* need to be investigated in the future.

### The *D11* is a novel dominant dwarf mutant

Four maize dominant dwarf mutants, including *D8*, *D8-1023*, *D9*, and *Dt*, have been characterized (Table 1). The *D8* and *D9* all display narrow and dark green leaves. The *D8-1023* is with broad, dark green, and pursy leaves. Leaves of *D11* appear slender, dark green, and slightly-rolled. Remarkably, *D11* possesses white leaf margins. Three maize dominant dwarf plants *D8*, *D8-1023*, and *D9* show tillering ability. Floral organs of *D9* are normal. Although *D8* and *D8-1023* display andromonoecious floral organs, they could be successfully self-pollinated. In contrast, *D11* has severe defects in inflorescence development. Commonly, tassel of *D11* is surrounded by multiple degenerate spikes which are almost barren. In extreme cases, female inflorescence dies aborning and no apparent ear is observed in *D11*. Thus, it is difficult to self-pollinate of *D11*. Compared with wild-type, *D11* flowers earlier. Similar result was observed in *D8* plant [9]. Intriguingly, maize transgenic experiment demonstrated that overexpression of *D9-1* delays flowering, while overexpression of *d9* promotes flowering [8]. Different dwarf genes may be recruited by distinct central modulators of complicated network to finely control flowering time in maize. Besides, *D8* and *D9* plants are insensitive to GA stimulation and belong to the GA-insensitive type [9], [23]. Conversely, *D11* responds to exogenous GA stimulation (Figure 2). In this study, transcriptomic analysis provides further evidence of *D11* as a GA-sensitive mutant (Figure 4).

Taken together, compared with other four maize dominant dwarf mutants (*D8*, *D8-1023*, *D9*, and *Dt*), *D11* is a novel dominant dwarf mutant, characteristic of dwarf stature, slightly-rolled leaf with white margins, deformed inflorescence, and GA sensitivity. Further gene cloning and functional characterization are informative for dissecting *D11*-mediated network in the control of plant stature and other developmental programs.

## Materials and Methods

### Plant Materials and Traits Evaluation

Maize inbred line Mo17, as female plant, was crossed with maize inbred line HN06 with normal plant height and unknown pedigree to generate F_1_ progeny. In F_1_ population, the dwarf mutant *D11* was identified. Then, *D11* as donor parent was continuously backcrossed with three maize inbred lines Mo17, B73, and W22 to develop advanced backcross populations BC_6_F_1_ with different nuclear background.

All experimental materials were single-grain sowed and nursed with normal agricultural practice. Traits were evaluated with ten consecutive plants starting from the third plant in each row. Plant height was measured from the ground to tassel top. For leaf number count, the fifth, tenth, and fifteenth leaves were firstly marked in red paint. Leaf number was counted when the tassel achieved fixed-length. Leaf area was calculated with the following formula: Leaf area  =  Leaf length × leaf width ×0.75. Days to pollen shed were measured as number of days from sowing to emergence of the first pollen from anthers in the central axis of the tassel.

### Sequence Polymorphisms Survey

Primers designed on 5′ and 3′ UTRs of *d8* and *d9* genes were used to amplify wild-type and *D11* DNA templates. Target amplicons were purified from gel with the QIAquick® gel extraction kit (Qiagen, Germany) and ligated into pGEM-T vector (Promega, USA) for sequencing (BGI, China).

### Physiological Characterization

Seeds of wild-type and *D11* plants (*n* = 35) were surface sterilized with a 2% NaClO solution for 20 min, washed by distilled water for five times. For germination, sterilized seeds were placed on filter paper which was saturated with distilled water or 10^−4^ M GA_3_ (Sigma, USA) solution. Germinated seeds were transferred to roseite for growth. Seedlings of WT and *D11* were sprayed with distilled water or 10^−4^ M GA_3_ solution per day. Ten days later, length of shoot, coleoptile, leaf blade, and leaf sheath was measured in WT and *D11*. For PAC treatment, sterilized seeds of wild-type and *D11* (*n* = 40) were firstly soaked in a distilled water or 10^−4^ M PAC (Sigma, USA) solution for 24 h, then embedded in distilled water for 24 h. Treated seeds were transferred to roseite for growth. Fifteen days later, shoot length was determined in WT and *D11*.

### Microarray Hybridization and DEGs Identification

The GeneChip® Maize Genome Array (Affymetrix, USA) was used for microarray analysis in this study. Firstly, total RNA was extracted from WT and *D11* stems by TRIzol® Reagent (Invitrogen, USA), following the manufacturer's instructions. RNA integrity was assessed by the Agilent Bioanalyzer 2100 (Agilent technologies, USA). Qualified total RNA was further purified by RNeasy micro kit (QIAGEN, Germany) and RNase-Free DNase Set (QIAGEN, Germany). Then, qualified total RNA were amplified, labeled, and purified using GeneChip® 3′IVT Express Kit (Affymetrix, USA) with the guidance of the manufacturer's manual to obtain biotin labeled aRNA. Array hybridization and wash were implemented using GeneChip® Hybridization, Wash, and Stain Kit (Affymetrix, USA) in Hybridization Oven 645 (Affymetrix, USA) and Fluidics Station 450 (Affymetrix, USA) according to the manufacturer's instructions. Slides were scanned by GeneChip® Scanner 3000 (Affymetrix, USA).

Raw data were submitted to the Gene Expression Omnibus database (accession number: GSE46370). Raw data were normalized by MAS 5.0 algorithm implemented in the Gene Spring Software 11.0 (Agilent technologies, USA). Normalized data of *D11* divided by those of WT obtained fold change. Genes whose transcripts were up-regulated or down-regulated at levels greater than a fivefold ratio were regards as DEGs in this study. Non-redundant DEGs were firstly annotated by the NCBI Entrez Gene resource (http://www.ncbi.nlm.nih.gov/gene). Then, DEGs were mapped on the B73 reference genome using the B73 sequencing database (http://www.maizesequence.org/index.html). Gene ontology (GO) enrichment analysis of DEGs was performed through the Gene Ontology (http://www.geneontology.org/) and agriGO (http://bioinfo.cau.edu.cn/agriGO/index.php) webservers [34].

### qRT-PCR Validation of DEGs

Some DEGs involved in GA metabolism were validated by qRT-PCR technique. Purified RNA samples used for microarray analysis were firstly transcribed to obtain cDNA by M-MLV reverse transcriptase (Promega, USA). A total of 10 µl qRT-PCR reaction contained cDNA template, 1×PCR buffer, 200 µM dNTPs, 0.2 µM primers, 0.5 U Taq DNA polymerase (Invitrogen, USA), and appropriate amount of distilled water. The mixture was predenatured at 94°C for 2 min, followed by 30 cycles of denaturation at 94°C for 30 s, annealing at 55°C for 30 s, and extension at 72°C for 30 s. The final extension step was set at 72°C for 10 min. Primers reported by Song et al. [35] were used for qRT-PCR analysis.

## Supporting Information

Figure S1 **Characteristics of** ***D11*** **leaf.** (**A**) Phenotype of the first, second, and third leaves (from top to bottom) of *D11* and wild type (WT). The *D11* mutant has slender leaves with white margins. Bar  = 5 cm. (**B**) Leaf number. The *D11* mutant possesses more leaves. (**C**) Leaf length and width. Compared with wild-type, leaves of *D11* are relatively long and narrow. (**D**) Ratio of leaf length to width. (**E**) Leaf area. Average values were calculated (*n* = 30). Data are mean ±SD. Single asterisk and double asterisks indicate significant difference at P≤0.05 and P≤0.01 levels compared with the wild type by Student's *t* test, respectively.
(TIF)

Figure S2 **PCR products amplified by primers designed on UTRs of** ***d8*** **and** ***d9*** **.** **M:** 1 kb marker; **Lanes 1 and 2:** amplicons from *d8* UTRs primers; **Lanes 3 and 4:** amplicons from *d9* UTRs primers.
(TIF)

Figure S3 **Response of** ***D11*** **to GA_3_ stimulation.** (**A**) Shoot length. (**B**) Coleoptile length. (**C**) The first leaf blade length. (**D**) The first leaf sheath length. (**E**) The second leaf blade length. Data are mean ±SD (*n* = 35). Double asterisks denote significant difference at P≤0.01 level compared with untreated samples by Student's *t* test.
(TIF)

Figure S4 **GO clustering of down-regulated DEGs.**
(TIF)

Table S1 **Segregation in backcross and self-pollination populations from** ***D11*** **and normal height plants.**
(DOC)

Table S2 **Primers used in this study.**
(DOC)

Table S3 **Detailed information of DEGs.**
(XLS)
