# Supplementary figures and images for: Gibberellin Biosynthetic Deficiency Is Responsible for Maize Dominant Dwarf11 (D11) Mutant Phenotype: Physiological and Transcriptomic Evidence
Source: PLoS One. 2013 Jun 12;8(6):e66466. doi: 10.1371/journal.pone.0066466 (PMC3680376; doi:10.1371/journal.pone.0066466)

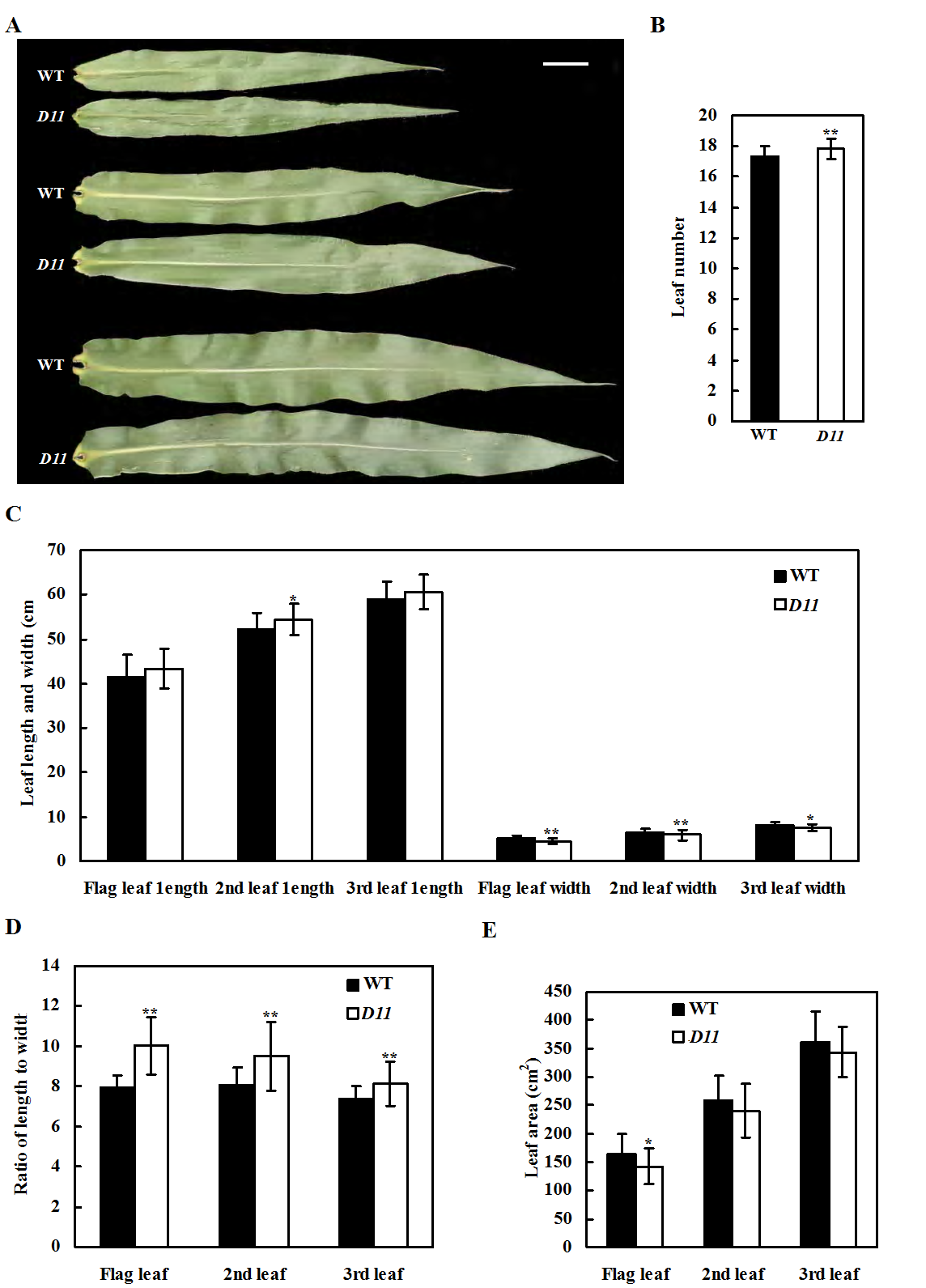

Supplement: Figure S1 — Characteristics of D11 leaf. (A) Phenotype of the first, second, and third leaves (from top to bottom) of D11 and wild type (WT). The D11 mutant has slender leaves with white margins. Bar = 5 cm. (B) Leaf number. The D11 mutant possesses more leaves. (C) Leaf length and width. Compared with wild-type, leaves of D11 are relatively long and narrow. (D) Ratio of leaf length to width. (E) Leaf area. Average values were calculated (n = 30). Data are mean ±SD. Single asterisk and double asterisks indicate significant difference at P≤0.05 and P≤0.01 levels compared with the wild type by Student's t test, respectively. (TIF) [file pone.0066466.s001.tif]

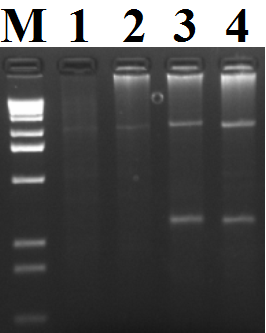

Supplement: Figure S2 — PCR products amplified by primers designed on UTRs of d8 and d9 . M: 1 kb marker; Lanes 1 and 2: amplicons from d8 UTRs primers; Lanes 3 and 4: amplicons from d9 UTRs primers. (TIF) [file pone.0066466.s002.tif]

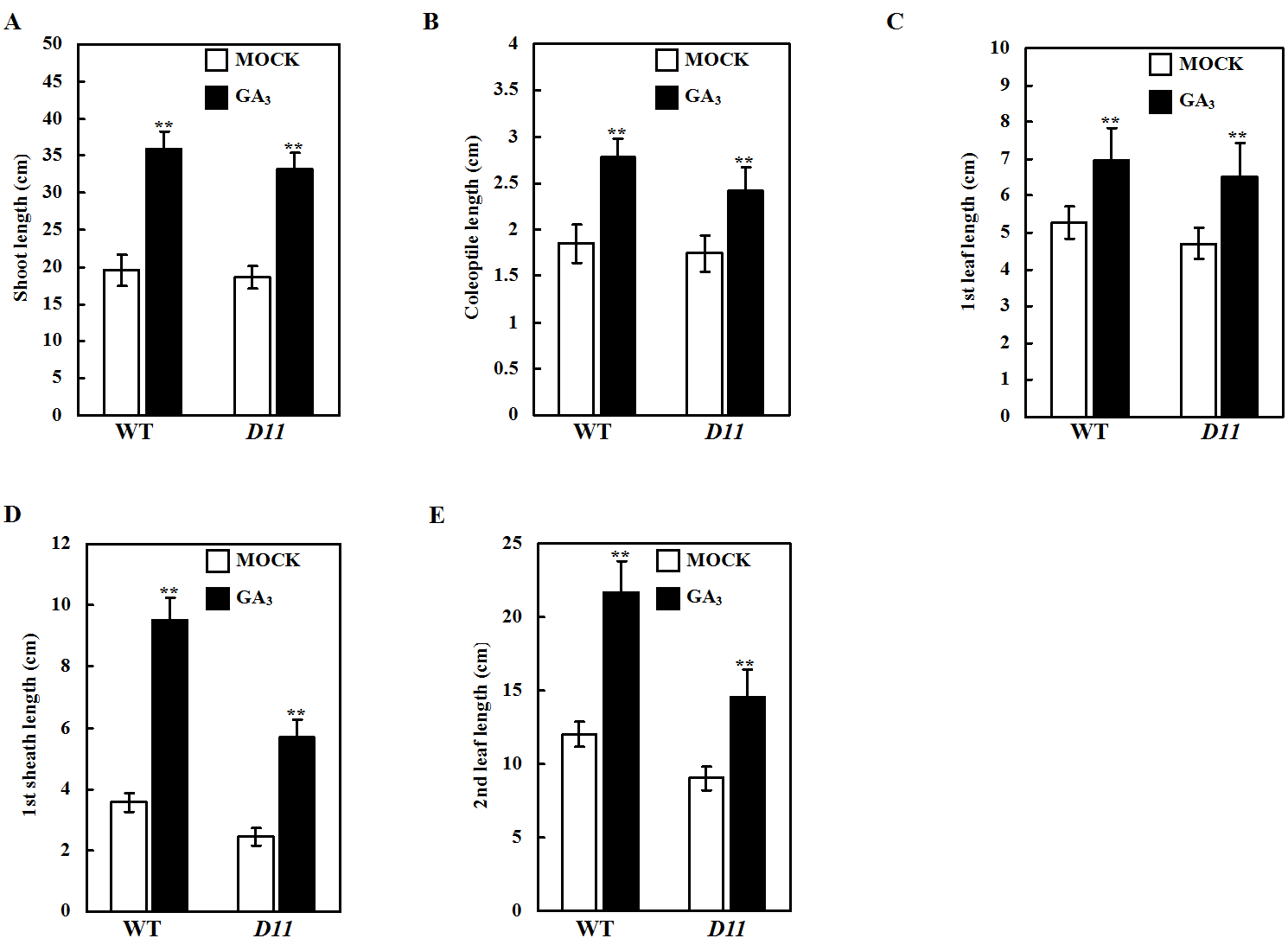

Supplement: Figure S3 — Response of D11 to GA3 stimulation. (A) Shoot length. (B) Coleoptile length. (C) The first leaf blade length. (D) The first leaf sheath length. (E) The second leaf blade length. Data are mean ±SD (n = 35). Double asterisks denote significant difference at P≤0.01 level compared with untreated samples by Student's t test. (TIF) [file pone.0066466.s003.tif]

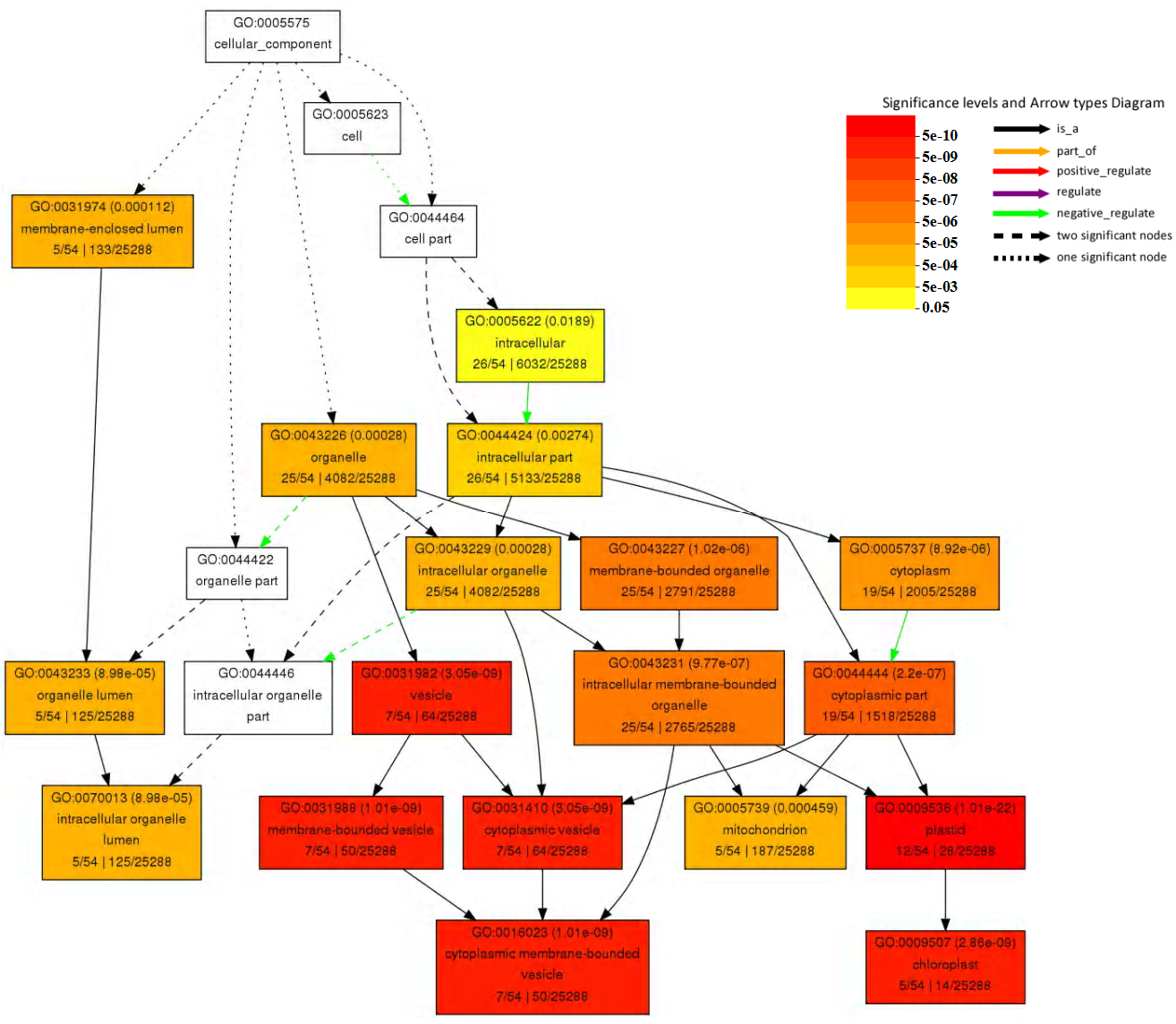

Supplement: Figure S4 — GO clustering of down-regulated DEGs. (TIF) [file pone.0066466.s004.tif]
