# Supplementary material for: Gibberellin Biosynthetic Deficiency Is Responsible for Maize Dominant Dwarf11 (D11) Mutant Phenotype: Physiological and Transcriptomic Evidence
Source: PLoS One. 2013 Jun 12;8(6):e66466. doi: 10.1371/journal.pone.0066466 (PMC3680376; doi:10.1371/journal.pone.0066466)
Supplement: Table S1 — Segregation in backcross and self-pollination populations from D11 and normal height plants. (DOC) [file pone.0066466.s005.doc]

**Table S1.** Segregation in backcross and self-pollination populations from *D11* and normal height plants.

| Cross | Year/ Region | No. of plants | | $\chi^{2}$(1:1) | *P* |
| --- | --- | --- | --- | --- | --- |
|  |  | Dwarf plant | Normal height plant |  |  |
| Mo17 (+/+) × *D11* (+/*D11*) | 2009/ Yangzhou | 84 | 93 | 0.36 | 0.75─0.50 |
|  | 2013/ Sanya | 98 | 91 | 0.19 | 0.75─0.50 |
| Normal height plant (+/+) ⮾ | 2009/ Yangzhou | 0 | 75 |  |  |
|  | 2013/ Sanya | 0 | 89 |  |  |

$$\chi_{0.05,1}^{2}=3.84$$
