# Supplementary material for: Gibberellin Biosynthetic Deficiency Is Responsible for Maize Dominant Dwarf11 (D11) Mutant Phenotype: Physiological and Transcriptomic Evidence
Source: PLoS One. 2013 Jun 12;8(6):e66466. doi: 10.1371/journal.pone.0066466 (PMC3680376; doi:10.1371/journal.pone.0066466)
Supplement: Table S2 — Primers used in this study. (DOC) [file pone.0066466.s006.doc]

**Table S2.** Primers used in this study.

| Trial | Gene | Region | Primer sequence |
| --- | --- | --- | --- |
| Sequence variation | *d9* | 5′ UTR | 5′–GCTGCTACTACTAGTTGCCTTGCTCGCTTC–3′ |
|  |  | 3′ UTR | 5′–GCAGCGTTCACTGACGTCTTAGCTTCCAC–3′ |
|  | *d8* | 5′ UTR | 5′–TTAGCTGGCTAGCTAGGCCTGT–3′ |
|  |  | 3′ UTR | 5′–CGTACGTGTGCCTTGATCGGCGTCCAGAAG–3′ |
| qRT-PCR | *ZmKAO* | Forward | 5′–TCTTCCTCCACCACTTCCTC–3′ |
|  |  | Reverse | 5′–CAGCCGCTAGAGTTTCTGCT–3′ |
|  | *ZmGA20ox1* | Forward | 5′–GCGCTACTGCTCTGAGATGA–3′ |
|  |  | Reverse | 5′–CACGGCGGGTAGTAGTTGAG–3′ |
|  | *18s rRNA* | Forward | 5′–ACATGCGCCTAAGGAGAAATAG–3′ |
|  |  | Reverse | 5′–ACCTCCATGCTCACTGGTACTT–3′ |
